# An unanticipated diagnosis with bedside ultrasonography in patients with acute abdominal pain: rectus hematoma

**DOI:** 10.11604/pamj.2017.27.19.12432

**Published:** 2017-05-08

**Authors:** Erden Erol Ünlüer, Eylem Kuday Kaykısız

**Affiliations:** 1 Emergency Department Usak University Department of Emergency Medicine Usak/Turkey; 2 Bitlis State Hospital, Department of Emergency Medicine, 13000 Bitlis/Turkey

**Keywords:** Abdominal pain, ultrasonography, hematoma

## Abstract

Although abdominal pain is a common presentation in emergency departments, rectus sheath hematoma (RSH) is among the rarest diagnosis. Here we present 2 cases of RSH likely caused by coughing due to upper respiratory tract infection. The two described cases were diagnosed by bedside ultrasonography and confirmed as RSH by computed tomography. Review of patient history and use of ultrasonography are important to avoid misdiagnosisof RSH.

## Introduction

Although incidence data is extremely limited, rectus sheath hematoma (RSH) is an uncommon cause of abdominal pain. Evidence suggests that a diagnosis of RSH, which is done by bedside ultrasonography (BUS), significantly alters subsequent clinical management. Early diagnosis of RSH may prevent invasive testing, such as laparotomy, and unnecessary hospitalization. Here we describe 2 patients with RSH that presented without any identifiable risk factors except persistent dry cough over a one week period, after which diagnosis was made by bedside ultrasonography.

## Patient and observation

**Figure 1:**
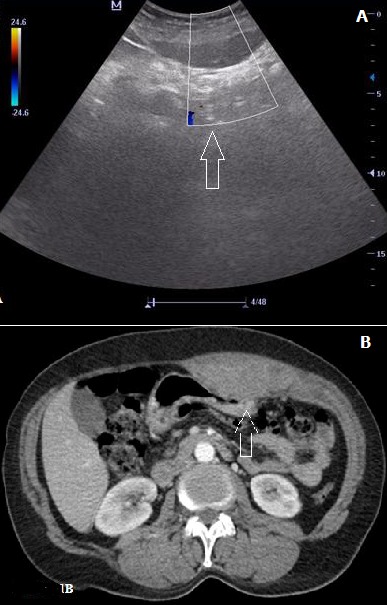
(A) bedside ultrasonographic view of the cystic mass in the anterior abdominal wall without blood flow; (B) enhanced CT scan showing a 3.5x4 cm fusiform dilatation in the left rectus muscle and diffuse increased density. Note the thickening of the abdominal wall

**Figure 2:**
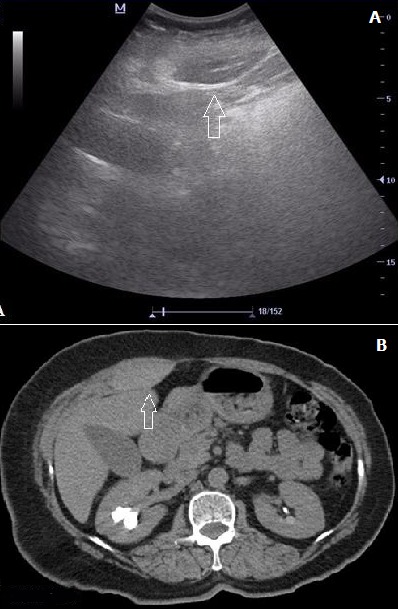
Bedside ultrasonographic view of the cystic mass in the anterior abdominal wall; (B) enhanced CT scan showing a 5.8x2.7 cm ovoid dilatation in the right rectus muscle and thickening of the abdominal wall muscles adjacent to the rectus

For the first case encountered at our emergency department, a 54 year old woman presented with abdominal pain, nausea and vomiting for 3 hours. She had no history of any chronic disease requiring regular drug usage, except a recent viral upper respiratory infection that persisted for 5 days with a dry cough. Physical examination found nothing unusual except tenderness of the left upper abdomen. Blood tests showed normal CRP, white blood cell count and blood chemistry results. Her hemoglobin was 14 g/L and INR was 1.04. Bedside ultrasonography (BUS) was performed by the emergency physician using a M5^®^ model ultrasound machine with a 5 MHz curved array probe (M5, Mindray Bio-medical Electronics CO, Shenzen, China). BUS showed a cystic mass in the anterior abdomen without blood flow Figure 1 (A). Abdominal computed tomography (CT) showed that the mass was a rectus sheath hematoma Figure 1 (B). For the second case, a 63 year old woman with a history of viral upper respiratory infection and cough for one week presented with abdominal pain for 2 hours. She had chronic hypertension and was taking medication for antihypertensive therapy. Once again, physical examination found nothing unusual except right upper abdominal tenderness. Her blood tests, including coagulation parameters, returned normal. Bedside ultrasonography was performed by the emergency physician as previously described, and a cystic mass was detected in the rectus muscle Figure 2 (A). As shown in the transverse CT of the right upper abdomen Figure 2 (B), abdominal CT confirmed diagnosis of rectus sheath hematoma. Both patients were discharged from the emergency department after controlled hemoglobin levels. Analgesic and antitussive therapy was ordered for out-patient follow up. Informed consent form was obtained from the patients at the time of diagnose.

## Discussion

Over the last two decades, interest in the development and use of BUS to assess patients admitted to the emergency department has grown considerably. Several studies on detection of hepatobiliary diseases or abdominal aortic aneurysm have investigated the application of BUS to assess the abdominal pain [1, 2]. During evaluation of abdominal pain by BUS, superficial structures may go undetected depending on the ultrasound operator's experience and the curved array probe used. A curved array probe is commonly used for ultrasonographic screening of the abdominal area. The low frequency and increased wavelength of this probe is not suitable for more superficial structures, such as the abdominal wall, and the operator may easily miss the superficial localized disease process. RSH is frequently misdiagnosed as appendicitis, cholecystitis, incarcerated inguinal hernia, torsion of ovarian cyst, or acute pancreatitis [3]. Predisposing factors include hypertension, obesity, previous abdominal surgery, coughing, straining, subcutaneous injections of heparin into the abdominal wall, and use of oral anticoagulants. RSH is usually managed conservatively with rest, analgesics, and discontinuation of any anticoagulant therapy when necessary. Surgical evacuation and hemostasis is necessary if the hematoma fails to resolve

## Conclusion

A careful review of patient history, high degree of suspicion together with thorough screening of superficial abdominal structures using BUS may aid incorrect diagnosis of RSH, preventing unnecessary exploratory laparotomies.

## Competing interests

The authors declare no competing interests.
